# A Review of Red Yeast Rice, a Traditional Fermented Food in Japan and East Asia: Its Characteristic Ingredients and Application in the Maintenance and Improvement of Health in Lipid Metabolism and the Circulatory System

**DOI:** 10.3390/molecules26061619

**Published:** 2021-03-15

**Authors:** Hiroyuki Fukami, Yuki Higa, Tomohiro Hisano, Koichi Asano, Tetsuya Hirata, Sansei Nishibe

**Affiliations:** 1 Central R&D Laboratory, KOBYASHI Pharmaceutical Co., Ltd., Ibaraki 567-0057, Japan; y.higa@kobayashi.co.jp (Y.H.); t.hisano@kobayashi.co.jp (T.H.); ko.asano@kobayashi.co.jp (K.A.); t.hirata@kobayashi.co.jp (T.H.); 2 Faculty of Pharmaceutical Sciences, Health Sciences University of Hokkaido, Ishikari 061-0293, Japan; nishibe@hoku-iryo-u.ac.jp

**Keywords:** red yeast rice, *Monascus* fungi, solid-state fermentation, monacolin, statin, LDL-cholesterol, polyketide, pigment, lipid metabolism, cardiovascular system

## Abstract

Red yeast rice has been used to produce alcoholic beverages and various fermented foods in China and Korea since ancient times; it has also been used to produce *tofuyo* (Okinawan-style fermented tofu) in Japan since the 18th century. Recently, monacolin K (lovastatin) which has cholesterol-lowering effects, was found in some strains of *Monascus* fungi. Since statins have been used world-wide as a cholesterol-lowering agent, processed foods containing natural statins are drawing attention as materials for primary prevention of life-style related diseases. In recent years, large-scale commercial production of red yeast rice using traditional solid-state fermentation has become possible, and various useful materials, including a variety of monascus pigments (polyketides) that spread as natural pigments, in addition to statins, are produced in the fermentation process. Red yeast rice has a lot of potential as a medicinal food. In this paper, we describe the history of red yeast rice as food, especially in Japan and East Asia, its production methods, use, and the ingredients with pharmacological activity. We then review evidence of the beneficial effects of red yeast rice in improving lipid metabolism and the circulatory system and its safety as a functional food.

## 1. Introduction: Overview of Red Yeast Rice, Its History, Production Method, and Use 

### 1.1. The History behind Koji (Yeast Grain) and Red Yeast Rice (Red Koji)

Every country in the world has its own traditional food culture, and quite a few countries have their own brewed foods, made using different fermentation mechanisms. In contrast to western countries, where malt and fruit juice are used for fermentation, in Eastern and Southeastern Asia, fermented foods are produced using rice and beans malted with filamentous fungus such as *Aspergilli.* Fungi are widely used since the areas have a climate of high temperatures and humidities.

*Koji* is made by breeding molds that can be used for food fermentation on grains such as rice, wheat, and soybeans. *Miso*, soy-sauce, vinegar, *mirin* (sweet *sake*), pickles, *sake* (Japanese rice wine), and distilled spirit are all made from various *Koji* grains which play a central role in “*Wasyoku*”, a food culture in Japan.

There are various types of *koji* and *koji* molds (also called *koji* fungi): yellow *koji* (made with *A. oryzae*), used to produce *miso*, soy-sauce, *mirin* and *sake*; black *koji* (made with *A. awamori*), used to produce *awamori* (Okinawan distilled spirit); and black *koji* or white *koji* (made with *A. Kawachii*), used to produce *syochuu* (Japanese distilled spirits). Red yeast rice (red yeast grain, red *koji*, *Beni-Koji*), made with *Monascus* fungi, has been used to produce *Tofunyu* (fermented bean curd) in Taiwan and China since ancient times and has also been used to produce *tofuyo* (Okinawan style fermented bean curd) in Japan. 

**Figure 1:**
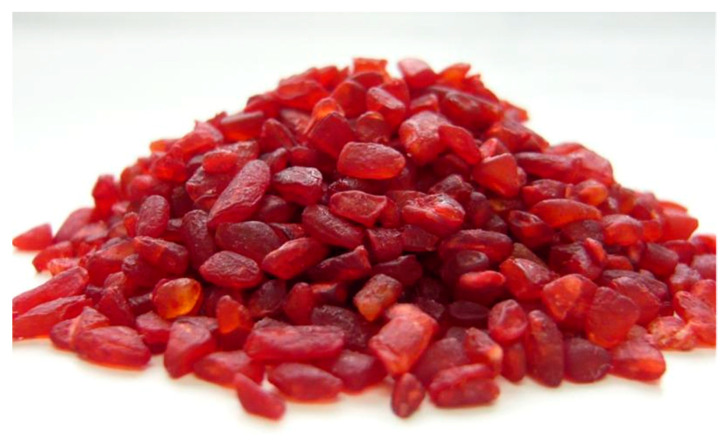
Appearance of red yeast rice.

*Monascus* fungi produce red pigments and the *koji* produced with *Monascus* fungi exhibit a deep red color (Figure 1). These red pigments have been used industrially since the 1950s in Japan. For example, they have been used in processed fish paste products, such as crab-flavored-fish cakes and processed meat products, such as ham and sausages. Recently, they have been widely used in various foods including bread, confectionaries and beverages, such as *amazake* (fermented rice drink). 

Red yeast rice is recorded in *Bencao Gangmu* (Compendium of Materia Medica), a representative book on Chinese medicine, written in 1596 which has been highly valued since ancient times in China. Lovastatin (monacolin K), which has cholesterol lowering effects, was found as an active ingredient from *Monascus* fungi [1,2,3]. Statins such as lovastatin and its analogs are used world-wide as serum cholesterol lowering agents which are used as first-line drugs for atherosclerotic diseases. Evidence for the beneficial and pleiotropic effects of statins has been reported, and further accumulation of evidence is expected in the future. Red yeast rice also contains lovastatin, and although it is currently used as a functional food, it could be used in the prevention of life-style related diseases to reduce metabolic syndrome and the metabolic domino, through the improvement of dietary habits. 

In this study, we review *Monascus* fungi: their history, production methods of red yeast rice, foods made using red yeast rice, characteristic ingredients, evidence of potential beneficial effects to improve lipid metabolism and the circulatory system, and safety.

### 1.2. Monascus Fungi and Red Yeast Rice

*Monascus* fungi are taxonomically classified into *Hemiascomycetes*; they are filamentous fungi belonging to the *Ascomycetes* class, similar to *Aspergillus,* which are used in the production of *miso*, soy-sauce and *sake*. These *Monascus* fungi are called red *koji* mold (or red koji fungi) because they produce red pigments; approximately 20 strains have been identified so far (Table 1). Most of the *Monascus* fungi were found in red yeast rice and brewed foods or beverages produced with red yeast rice in China, Taiwan, or Korea from the second half of the 1920s to the first half of the 1930s [4]. About 50 strains of *Monascus* fungi are preserved in the Biological Resource Center, Department of Biotechnology, National Institute of Technology and Evaluation (NITE) in Japan (Table 2) [5].

### 1.3. Production of Red Yeast Rice

**Figure 2:**
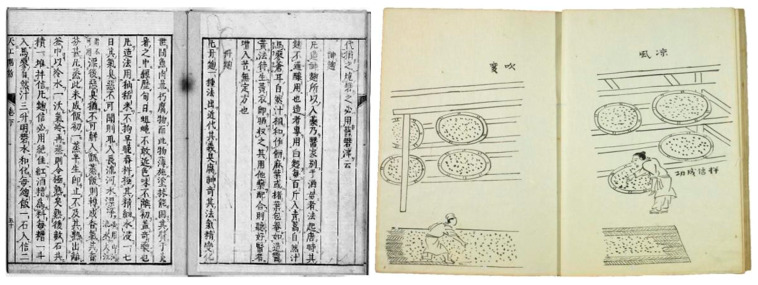
Production method of red yeast rice described in “*Tiangong Kaiwu*”. Rice inoculated with *Monascus* fungi is divided between several bamboo trays, which are placed on shelves to maintain good air circulation. Ambient air during the culture is a key factor. The room in which the shelves are placed must be wide and there must be a high ceiling. The room must also be facing south to avoid afternoon sunlight and its temperature should be controlled. Rice inoculated with the fungi should be mixed up and down 3 times every 2 h.

Red yeast rice is documented as Chinese herbal cuisine that improves blood circulation in *Nichiyo-Honzo,* a Chinese herbology book written by Duan Wu in 1329. Red yeast rice was also called *tan-giku,* and its production method is described in *Tiangong Kaiwu*, a compendium on industry, agriculture and artisanry, written by Song Ying in the 17th century in China (Figure 2) [4,6]. In brief, after soaking in water for 7 days, polished rice is steamed, and the lees of Shaoxing wine are added as seed fungi and cultured in a tile room for about 1 week. Interestingly, the method is very similar to that used in the production of yellow *koji* in Japan. *Monascus* fungi (red *koji* fungi) have low proliferative ability, and about 1 week is required for the production of red yeast rice, though only 2 days are required for that of yellow *koji*. The long-term culture increases the chance of contamination by various germs; therefore, the area of each production process is kept thoroughly clean, and the aseptically cultured red yeast rice is used as the seed fungus.

Red yeast rice itself has been used in the coloring of foods, meat in particular, in China and Taiwan since ancient times. Red pigments extracted and isolated from red yeast rice have been produced as natural pigments on an industrial scale since 1945. Since the carcinogenicity of synthetic red pigments was discovered, the consumption of natural pigments (red yeast rice pigment) made by *Monascus* fungi has increased. 

**Figure 3:**
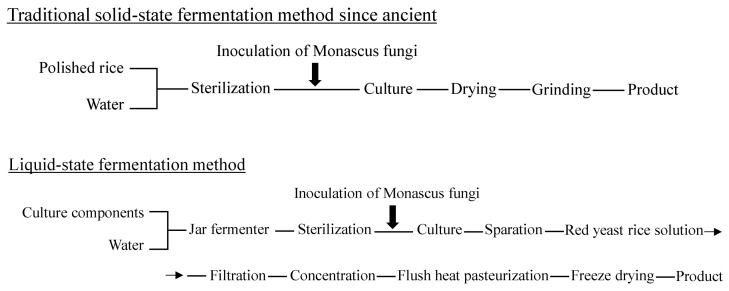
Solid-state/liquid-state culture method of red yeast rice.

The traditional method for the production of red yeast rice-related foods was the solid-state culture method described above; however, the pigments are now industrially produced by simple processes of extraction and concentration from red yeast rice, proliferated by an aerated and agitated culture method (liquid-state culture) (Figure 3) [7].

### 1.4. Brewed Foods Produced Using Red Yeast Rice

Red yeast rice has been widely used as a material for the production of *anchu* (Chinese red wine), *tofunyu (fermented bean curd)*, and a coloring agent for foods or a preservative for meat in China and Taiwan.

The production technique for red *Laojiu* (Chinese alcohol beverage) originated in a Fijian Province in China and was introduced into Taiwan about 200 years ago. It is said to be the origin of the present-day Taiwanese red alcoholic beverage called *Hon-ru-chu* that is popular at festivals and wedding ceremonies [4].

*Tofunyu*, also called *funyu* or *nyuhu*, is a flavored food produced using mold proliferated on bean cakes which are salted and then matured by soaking in unrefined *sake* [8,9]. Fermented bean curd produced using red yeast rice is often called red *funyu*, and it is said to have originated the period about 1500 years ago in China. Red *funyu* is now produced in Jiangsu, Zhejiang, Sichuan, Hong Kong and Taiwan. 

**Figure 4:**
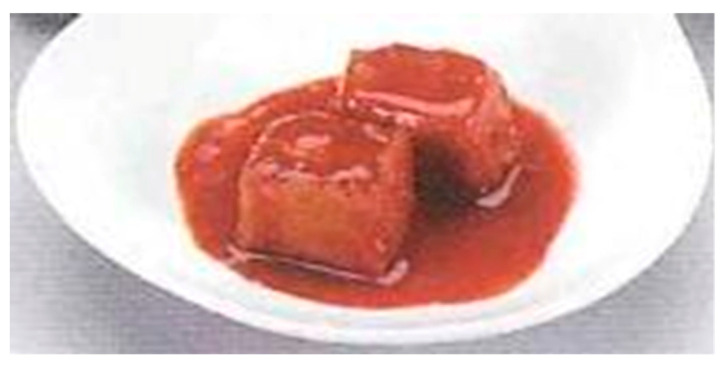
*Tofuyo*, a food that has been passed down the generations in Okinawa, Japan.

Brewed foods in East Asia were introduced to Japan sometime long ago. *Tofuyo,* a type of fermented bean curd (Figure 4), is a fermented food from Okinawa which is similar to red *funyu* in China and Taiwan. *Tofuyo* was introduced to Japan as “a fragrant and sweet/tasty food that improved digestive function and was effective in the treatment of various diseases” according to *Gyozen Honzo* (Edible plants of Okinawa), a book on dietary plants compiled in 1832 [4]. It was highly valued as a nutritional food and side dish in the 1800s. At present, *tofuyo* is produced by a novel method using aged *sake*, *awamori* (Okinawan distilled spirit).

*Funyu* has been eaten for more than 1000 years in China. In Japan, it is prepared using *miso* produced with red yeast rice. In 1985, a *Monuscus* fungi was used to brew *miso* prepared by a traditional method, and it has been in use ever since [10].

*Anchu* has been produced in Okinawa since the 18th century, according to a written record and products list [11]. For many years, it has been produced in China using yeast rice on which *Monascus* fungi are proliferated. During 1850–1900, it is said, red rice and steamed red rice cakes wrapped in bamboo leaves were a traditional Okinawan food [12]. In China, a book called “*Honzo-Jyushin*”, published in 1751, described a method for preparing red rice cake as follows: rice was mixed with red yeast rice and steamed, and the resultant red rice cake could be offered for food. Red rice is prepared by steaming rice grains after they have been mixed with red yeast rice. Red rice cakes are prepared by mixing red yeast rice soaked in *sake* and mashed in advance. The red yeast rice pigments mentioned above have been used in processed fish paste products (including crab-flavored fish paste cakes), jam, tomato ketchup, sweetened bean jam, stewed octopus, salmon roe, and processed meat products such as ham and sausage. In addition, red yeast rice pigments are used for coloring bread, confectionaries including rice confectionaries, and beverages, including *amazake*, and they are also widely used as pigments for foods in Asia and Europe [6,13].

## 2. Polyketides and Other Metabolites Produced in Monascus Fungi 

### 2.1. Polyketides Identified in Red Yeast Rice

The compendium “*Tiangong Kaiwu*” describes fish meat as “generally spoilable, but its quality can be kept by smearing red yeast rice on its surface, even in hot conditions; no fly or maggot will approach, even 10 days later: red yeast rice is a truly miracle agent”. This description indicates that red yeast rice has sterilizing or bacteriostatic action to prevent deterioration of fish meat caused by bacterial contamination (it may prevent fly swarming by other mechanisms). The custom to use red yeast rice in the preservation of foods is still used today; it is often used in the preservation of pork and chicken in Taiwan. Recent studies have demonstrated that *M. purpureus* produces substances with antibacterial activity against *Bacillus*, *Streptococcus,* and *Pseudomonas* species. Some of these substances are considered to be pigments. “*Bencao Gangmu”* (Compendium Materia Medica, vol. 25) written by Li Shizhen in 1590 describes a number of medicinal effects of red yeast rice, including that red yeast rice improves blood circulation, helps digestion, activates splenic function, cures diarrhea, heals bruises and injuries, enhances blood health, and protects a woman just after childbirth from blood stasis. 

**Figure 5:**
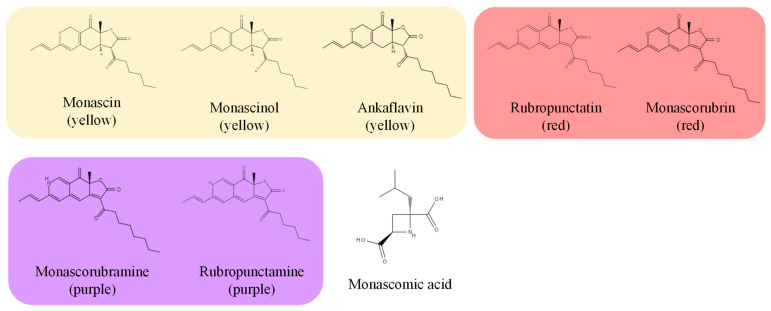
Major pigments (polyketides or azaphilone pigments) produced by *Monascus* fungi.

Red yeast rice pigments (polyketides) are called azaphilone pigments, and the following have been isolated and identified: monascin, ankaflavin, and monascinol (yellow pigments); rubropunctamine and monascorubramine (purple pigments); and rubropunctatin and monascorubrin (red pigments) (Figure 5) [4,7,14]. Furthermore, it has been reported that red yeast rice produces polyketide derivatives such as monascumic acid and γ-amino butyric acid (GABA) etc. [15,16,17]. Monascus pigments are known to have antibacterial activity and anti-cancer effects and GABA to have blood pressure-regulating effect [18,19,20]. It has also been reported that fermented rice bran with Monascus increases phenolic acid and enhances antioxidant activity [21,22]. In addition, organic acids, amino acids, sterols, decalin compound derivatives, flavonoids, lignans, coumarins, terpenoids, and polysaccharides are reported as components contained in red yeast rice [23].

**Figure 6:**
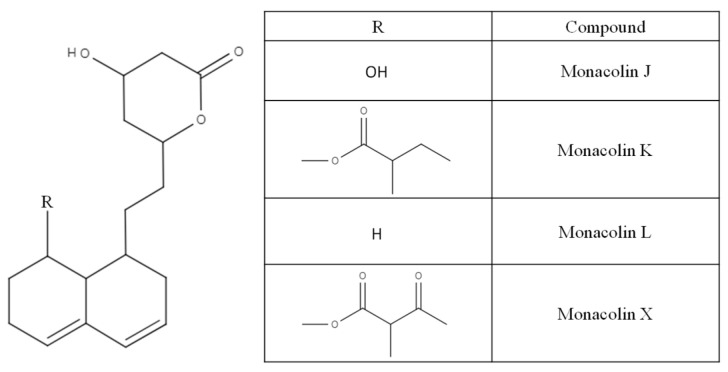
Monacolins (polyketides) produced by *Monascus* fungi.

**Figure 7:**
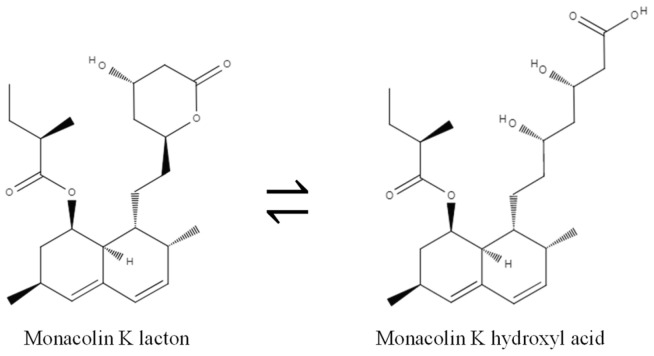
Lactone and acid forms of monacolin K.

A potent inhibitor of cholesterol synthesis, monacolin K (lovastatin) was discovered in 1979 in a strain of *Monascus* by Endo et al. [1,2,3,24]. Other active substances with a similar structure to monacolin K have since been found (Figure 6) [25,26]. Monacolins have two forms, acid or lactone form, depending on the conditions of the solution, particularly the pH (Figure 7) [27].

### 2.2. Production of Metabolites, Including Representative Polyketides in Traditional Solid-State Fermentation

Endo et al. also studied on red yeast rice that contained natural statins and favored by the Japanese in their diet. They examined color, taste and fragrance of various red yeast rice. Useful strains of *Monascus* fungi were bred, based on productivity of red yeast rice pigments and monacolin K. Recently, many attempts on breedings of Monascus strains and production conditions for efficient production of monacolin K have been reported [28]. The developed red yeast rice was produced using a traditional solid-state fermentation method [7].

**Figure 8:**
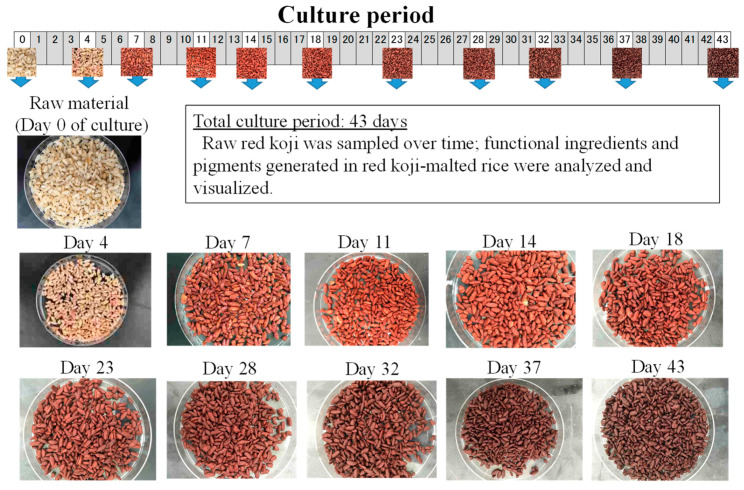
Time course change in appearance of red *koji*, which reddens during fermentation.

**Figure 9:**
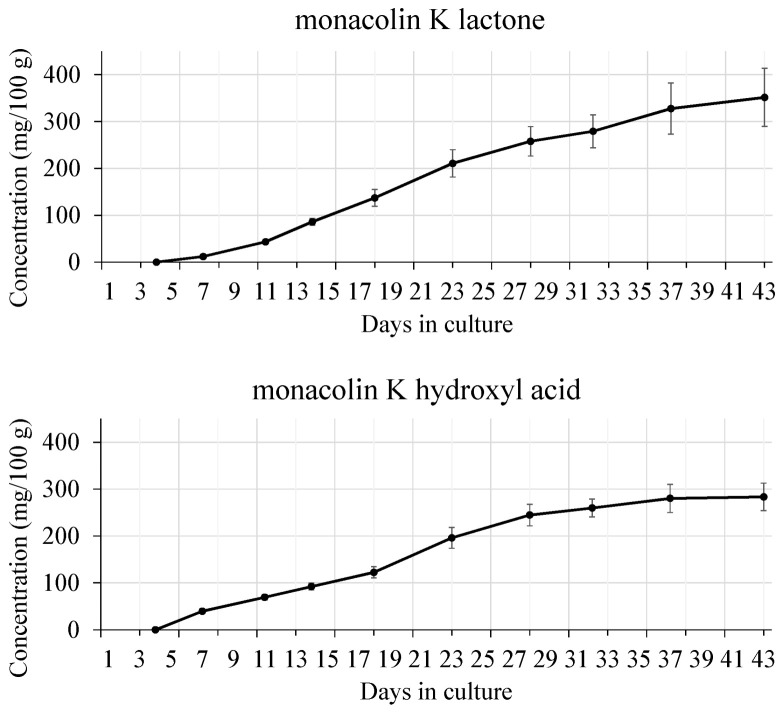
Time course change in the content of lactone form and acid form of monacolin K in red yeast rice during fermentation, quantified using the method specified by the Korea Food and Drug Administration (KFDA) [29].

**Figure 10:**
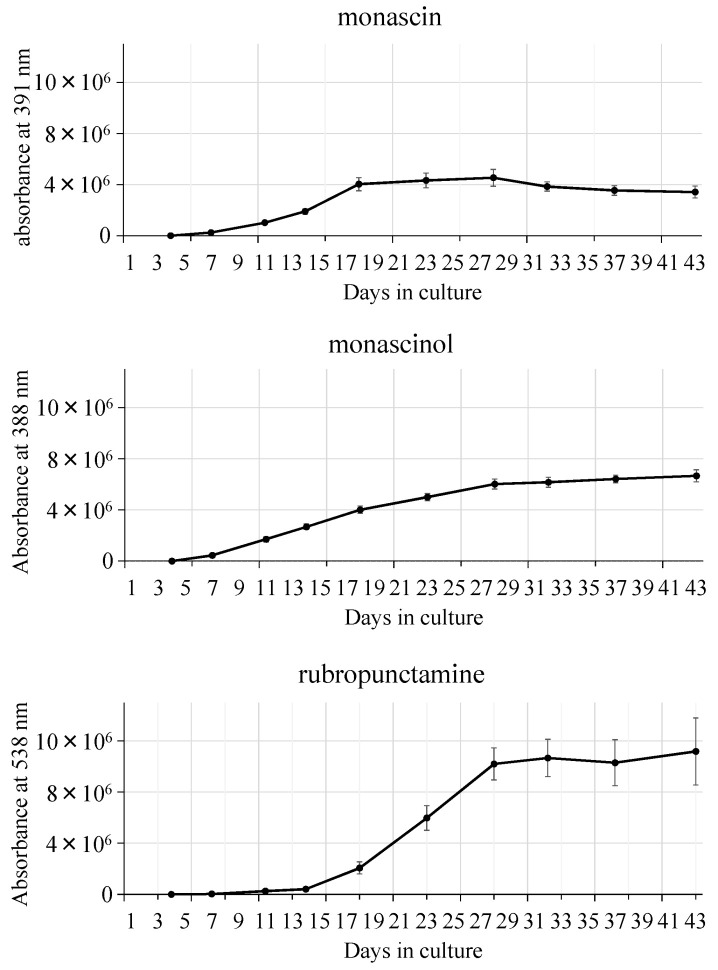
Time course change in the contents of 3 azaphilone pigments in red yeast rice during fermentation, quantified according to the reference method [30].

**Figure 11:**
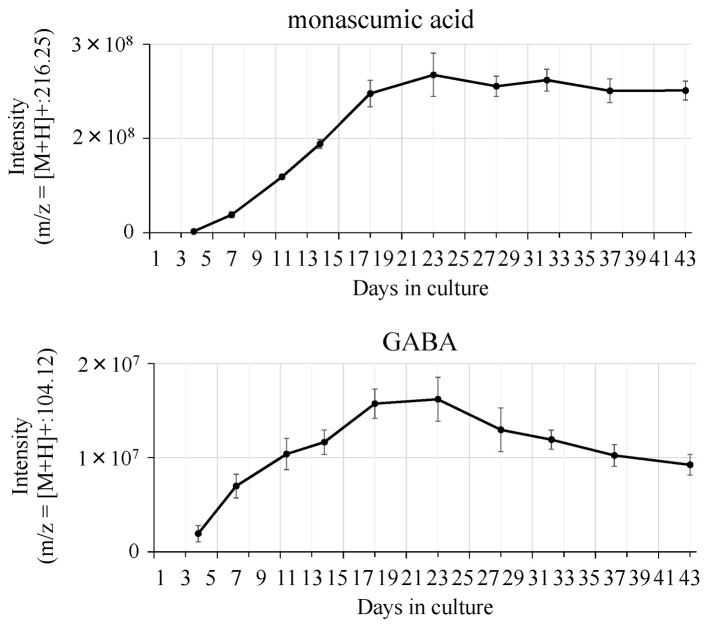
Time course change in the contents of 2 amino acids (GABA and monascumic acid) in red yeast rice during fermentation, quantified by liquid chromatography-mass spectrometry (LC-MS) [16,17].

*M. pilosus* is frequently used as a health food in Japan; we studied changes in its metabolite contents during growth. Steam-sterilized rice was inoculated with *M. pilosus* NITE BP-412, and a solid-state culture was started when the water content reached 42%. The culture was grown for 43 days; a temperature of 30 °C was used for the first 4 days and then 22 °C for the remaining time; the change in the contents of metabolites was then analyzed. The change in appearance of the red yeast rice during the 43 days is shown in Figure 8 and demonstrates the gradual change in color of the rice from white to red. The change in monacolin K content in the red yeast rice during fermentation was measured: The contents of both the lactone and acid form time-dependently increased (Figure 9). The contents of three azaphilone pigments (monascin, monascinol, and rubropunctamine) in red yeast rice were measured: The monascin content reached its peak 20 days after the start of the culture. Monascinol reached its peak 30 days after the start of the culture, and rubropunctamine began to increase about 10 days after the start of the culture and reached its peak at about 30 days. All pigments plateaued after they peaked (Figure 10). The time course change in the contents of two amino acids (GABA and monascumic acid) in red yeast rice during fermentation was also examined. The GABA content increased, with the peak occurring about day 20, and the level gradually decreased thereafter. The monascumic acid content also increased, with the peak on day 20, and the level plateaued thereafter (Figure 11).

The results suggest that *M. pilosus* fought against foreign enemies, utilizing monascumic acid as its antibacterial activity in the first half of their growth and monacolin K with its cholesterol synthesis inhibiting activity in the second half of their growth. GABA could be used as a nitrogen source in the second half of growth, when nutrients become deficient; however, we suggest GABA was mainly used to fight against foreign enemies since it decreased in the middle of the second half of the growth phase. Based on these findings, we expect further improvements to traditional solid-state fermentation methods will lead to the development of useful materials in the production of red yeast rice.

## 3. Effectiveness of Red Yeast Rice: Its Functional Ingredients and Physiological Actions on Lipid Metabolism and the Circulatory System 

### 3.1. Monacolins and Their Cholesterol Lowering Effect

**Figure 12:**
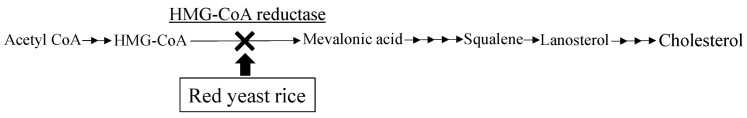
Biosynthetic pathway of cholesterol synthesis and the action of red yeast rice in it.

Monacolins are inhibitors of 3-hydroxy-3-methylglutaryl-CoA (HMG-CoA) reductase, a key enzyme regulating hepatic cholesterol synthesis, and exert potent serum cholesterol lowering effects (Figure 12). 

Lovastatin and its analogs (including their derivatives) have been used as medicines since 1987 by Japanese and foreign pharmaceutical companies. Statins inhibit HMG-CoA reductase, lower hepatic cholesterol biosynthesis and elevate the expression of hepatic LDL-receptors to maintain cholesterol homeostasis, which in turn enhances uptake of LDL-cholesterol into the liver and decreases blood cholesterol levels [31,32,33]. LDL-cholesterol is known as bad cholesterol because it is involved in atheroma formation and causes atherosclerosis. Since statins have excellent LDL-cholesterol-lowering effects, they are used as first-line drugs for patients with hypercholesterolemia, and several world-wide large-scale clinical trials have been conducted on statins. These trials have demonstrated not only their efficacy on cardiovascular disease such as cardiac infarction and cerebral stroke but also various actions such as potential preventive action on bone fracture. Statins prevented cardiovascular events by 25–30% in clinical trials assessing primary and secondary preventive effects on coronary artery disease, indicating that they are essential medicines for patients with cardiovascular disease [34,35]. Since it has been reported recently that statins show pleiotropic effects, such as a restorative effect on vascular endothelial functions and anti-inflammatory effects, an accumulation of evidence for their therapeutic and preventive effects on various diseases is expected [36,37].

Clinical studies in Japan have also been conducted on processed food containing red yeast rice produced using the traditional solid-state fermentation described in Section 2.2 [38,39,40].

**Figure 13:**
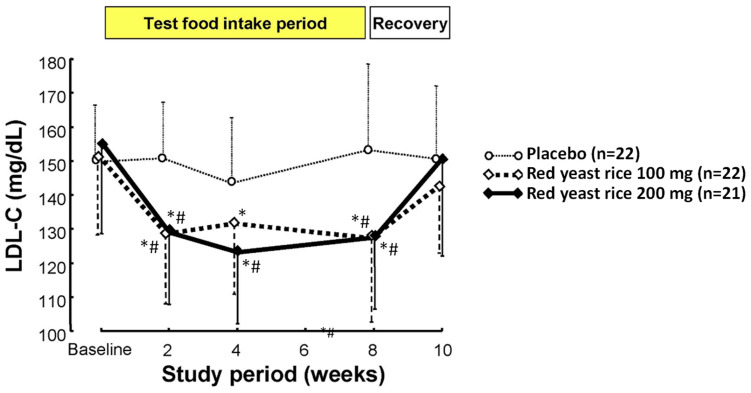
Effect of intake of red yeast rice produced by solid-state fermentation in normal healthy volunteers whose plasma cholesterol levels were 120 mg/dL or higher. Each bar indicates the mean ± standard deviation. *: Significant difference from start of ingestion (*p* < 0.05, paired ANOVA). #: Significantly different from the placebo group (*p* < 0.05, multiple comparison using Bonferroni).

In normal healthy volunteers with plasma LDL-cholesterol levels of 120 mg/dL or higher, long-term feeding of a processed food product containing 100 or 200 mg of red yeast rice produced by solid-state fermentation significantly lowered plasma levels of LDL-cholesterol and total cholesterol compared to those in volunteers fed the placebo at 2–8 weeks after starting the feeding. The lowered levels returned to the initial values after the end of red yeast rice intake. No significant differences in HDL-cholesterol and triglyceride levels and safety evaluation items among the 3 groups were found (Figure 13) [38]. In addition, in patients with hyperlipidemia where it was not improved during 1-month-diet therapy according to an NCEP panel, repetitive intake of a processed food product containing red yeast rice at 1 g/day significantly lowered levels of total cholesterol and LDL-cholesterol by about 10% [40]. Reports of clinical trials in Europe also show significant improvements in hypercholesterolemia by ingestion of red yeast rice containing 3–10 mg of monacolin K [41,42].

**Figure 14:**
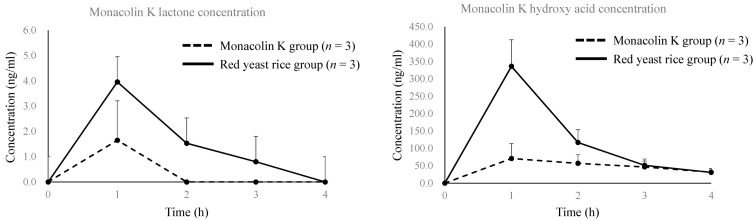
Plasma concentrations of lactone form and acid form of monacolin K after single administration of purified monacolin K or red yeast rice (40 mg/kg monacolin K or dose of red yeast rice equivalent to 40 mg/kg monacolin K) in male SD rats (7 weeks old). Each bar indicates the mean ± standard deviation, quantified according to the reference method [43].

Recently, we compared the pharmacokinetics of monacolin K in blood after oral administration of purified monacolin K (lactone form, 99% purity) and red yeast rice produced by solid-state fermentation in male SD rats (7 weeks old). Most of the monacolin K in the plasma was detected in its acid form: both in the monacolin K-administered group and the red yeast rice -administered group. Surprisingly, the maximum level (C max) and area under curve (AUC) of plasma monacolin K concentration 4 hours after administration were several times higher in the red yeast rice-administered group than in the purified monacolin K-administered group (Figure 14, Table 3). These results suggest that red yeast rice contains ingredients that enhance the absorption of monacolin K into blood. The medicinal benefit of red yeast rice is considered to be different to that of monacolin K alone. Furthermore, our recent experiments show that monacolin L, monascinol, and monascodilone also have HMG-CoA reductase activity. Since red yeast rice contains various ingredients, synergistic effects are expected.

### 3.2. Improving Effects of Red Yeast Rice on Chyle in Blood and Increased Viscosity Caused by Hyperlipidemia

**Figure 15:**
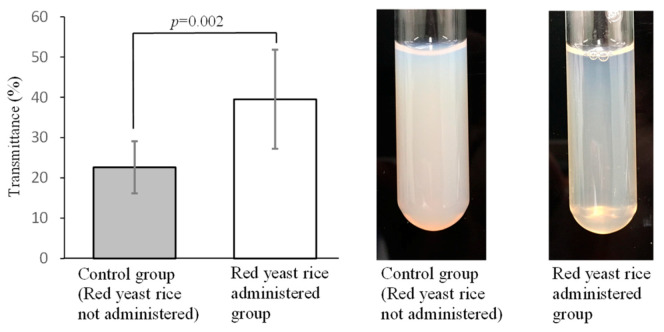
Difference in transmittance of plasma between red yeast rice-treated (2 weeks) and non-treated groups of male Japanese white rabbits (10–12 weeks old) fed a high-cholesterol diet. A decrease in turbidity in the red yeast rice-treated group is apparent. Each bar indicates the mean ± standard deviation. Significant differences were tested by Student’s *t*-test. Plasma cloudiness was evaluated by measuring the transmittance at a wavelength of 660 nm using an absorptiometer.

In general, continuous intake of a high-fat or high-cholesterol diet causes hyperlipidemia and the blood develops chyle, a white cloudy substance which mainly consists of lipoproteins. Chylemia is regarded as a risk factor for circulatory diseases, and its suppression is considered to be important for risk reduction. Recently, we conducted an experiment in which male Japanese white rabbits (10–12 weeks old) were fed a diet containing 0.25% cholesterol for 2 weeks to produce chylemia. The rabbits were then allocated into a red yeast rice-treated group, in which powdered red yeast rice was orally administered for 3 weeks or non-treated group. Blood samples were collected from both groups for plasma biochemical examinations and measurement of plasma turbidity. Macroscopic observations showed lower plasma turbidity in the red yeast rice-treated group compared with the non-treated group, and biochemical analysis revealed a statistically significant increase in light transmittance (Figure 15) and decrease in plasma total cholesterol levels in the red yeast rice-treated group compared with the non-treated group.

**Figure 16:**
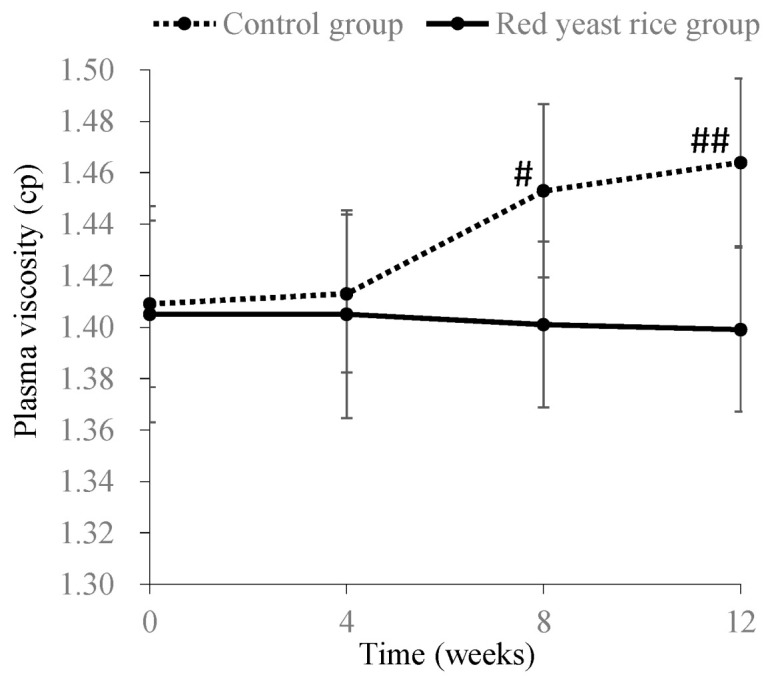
Time course changes in plasma viscosity in a control group and a red yeast rice-treated group in hypercholesterolemic model rabbits. Each bar indicates the mean ± standard deviation. Significant differences between control group and red yeast group were tested by Student’s *t*-test (#: *p* < 0.05, ##: *p* < 0.01). Plasma viscosity was measured using a plate and cone viscometer according to the reference method [50].

**Figure 17:**
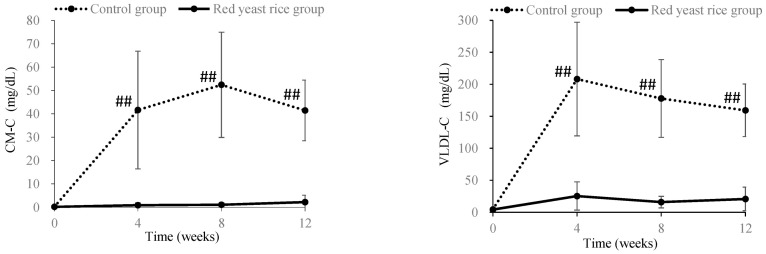
Time course change in contents of cholesterol in plasma low-density lipoproteins (chylomicron-cholesterol and VLDL-cholesterol) obtained from a red yeast rice-treated and control groups of hypercholesteremic model rabbits. Each bar indicates the mean ± standard deviation. Significant differences between control group and red yeast group were tested by Student’s *t*-test (##: *p* < 0.01). Plasma lipoprotein cholesterol was quantified according to the reference method [51].

It has also been reported that hyperlipidemia induces metabolic abnormality of lipoproteins and an increase in plasma viscosity, which in turn elevates the risk of cardiac and cerebrovascular diseases [45,46,47]. Lovastatin or ezetimibe, anti-hyperlipidemic drugs, have been reported to decrease plasma viscosity [48,49]. In our study, male Japanese white rabbits (14 weeks old) were fed a diet containing 0.25% cholesterol (HC diet) for 3 months as a hyperlipidemic animal model. During the 3-month feeding period, the control group was fed the HC diet alone, and the red yeast rice-treated group was fed the HC diet with red yeast rice at 500 mg/kg body weight. Blood biochemical analyses, performed during the feeding period, included measurements of plasma turbidity, viscosity and cholesterol content of lipoproteins. Plasma turbidity was improved, and total cholesterol and LDL-cholesterol plasma levels were reduced in the red yeast rice-treated group between months 1–3 of the experimental period, when compared to the control group. Furthermore, 3 months after the start of administration, the low-density cholesterol content of lipoproteins and plasma viscosity were still lower in the red yeast rice-treated group compared with the control group (Figure 16). In particular, the cholesterol contents in chylomicrons and VLDL were significantly lowered (Figure 17). These results indicate that ingredients in red yeast rice suppressed the increase in plasma viscosity by decreasing the content of large (low-density) lipoproteins through a reduction in VLDL release and clearance of chylomicrons.

Red yeast rice has the potential to prevent vascular disease associated with hyperlipidemia or reduce the risk for these diseases, through suppression of atheroma formation and improve blood fluidity, by lowering levels of chylomicrons and VLDL, lipoproteins that are the main components of plasma chyle.

### 3.3. Promising Effects of Ingredients in Red Yeast Rice on Risk Reduction for Circulatory Diseases

The evidence presented above suggests red yeast rice could reduce the risk of vascular diseases associated with hyperlipidemia; however, red yeast rice also contain statins and various other ingredients which are effective in the circulatory system. Table 4 summarizes the potential effects of monacolin K (lovastatin) on circulatory diseases, including cardiac disease. There has been a lot of evidence reported on the favorable actions of statins, including their HMG-CoA reductase inhibiting activity which suppresses atheroma formation in atherosclerosis [52,53,54], improving effects on blood circulation [55,56], and pleiotropic effects on nitric oxide (NO) production which plays a pivotal role in homeostasis of the cardiovascular system and the NO synthetase–NO pathway [57,58,59]. Effects of lovastatin on atherogenic signaling cascades [60] have also been reported [61,62,63]. There have also been similar favorable reports of red yeast rice extracts, such as upregulation of constitutive NO synthetase expression, the increase in plasma levels of nitric oxides and improvement of abnormal hemorheology in an atherosclerotic rat model induced by high-cholesterol diet feeding [64]. These effects are considered to be beneficial in the prevention of circulatory diseases and improvement in blood circulation.

Ingredients that are known to be effective in red yeast rice, other than statins, include GABA, which exerts a hypotensive effect through reduction of adrenergic vasoconstriction by its suppressive effect on the release of sympathetic nerve transmitters mediated by presynaptic GABA receptors [65,66]. In addition, GABA promotes sodium excretion from the kidneys and suppresses increases in blood pressure [67,68].

Oxidative stress is also regarded as a therapeutic target related to atherosclerosis. Reactive oxygen inactivates NO and at the same time damages vascular endothelial cells and reduces NO production. It has also been suggested that plasma LDL suppresses NOS activity in vascular endothelial cells by hydroxyl radicals and the like, thereby reducing NO production and promoting arteriosclerosis. [69,70]. Lovastatin inhibits oxidation of LDL, and this improves the vascular endothelial function (vasoconstrictor reaction) in hyperlipidemia, and in particular, the combined use of an antioxidant can significantly improve the function [64,71]. Antioxidant activity has also been reported in red yeast rice pigments [72,73]: monascin and ankaflavin suppressed LDL-cholesterol oxidation [74]. In addition, a previous clinical study reported that a red yeast rice extract reduced plasma levels of oxidized LDL, an index of oxidative stress, and lipoprotein-associated phospholipase A_2_ activity [75]. Red yeast rice made from brown rice, black rice, and red rice has also been shown to improve vascular endothelial function with increased NO production [76].

Monascin and ankaflavin are reported to act as the agonist for peroxisome proliferator-activated receptors (PPARs), e.g., PPARα [77]. PPARs that are involved in metabolism of carbohydrates, lipids, and energy are also closely involved in development of life-style related diseases, such as obesity and atherosclerosis. PPARα agonists (fibrates) have been clinically used as antihyperlipidemic drugs.

**Figure 18:**
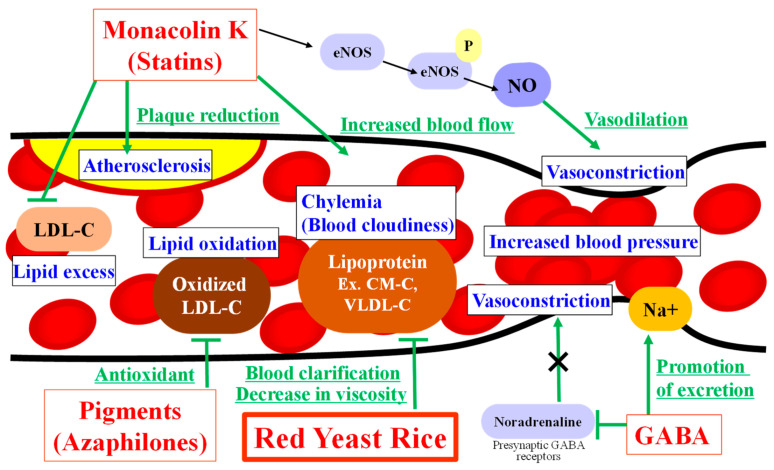
Expected effect on the circulatory system of red yeast rice and its components.

Vascular function can be improved by increasing NO, suppressing oxidative stress, hypertension, obesity, etc. [78,79,80]. It is expected that ingredients contained in red yeast rice, such as monacolin K (lovastatin), GABA, and red yeast rice pigments (azaphilone pigments) exert synergic or additive effects to lower the risk for atherosclerosis and other circulatory diseases (Figure 18). 

## 4. Safety of Red Yeast Rice

Red yeast rice produced properly by traditional solid-state fermentation using *Monascus* fungi is considered to be safe for food use; not only because of its long history within the Asian diet but also because red yeast rice manufacturers have made every effort to ensure its safety using various tests, including mutagenicity, acute toxicity, and chronic toxicity tests. The processed food containing red yeast rice used in a previous clinical trial has been commercially used for more than 20 years in Japan, and no problematic cases have been reported from a safety point of view. Furthermore, since red yeast rice pigments industrially produced with *Monascus* fungi are approved as food additives, their safety is institutionally secured by various toxicity tests based on legal standards [7].

Of the *Monascus* fungi, *M. pilosus*, *M. ruber*, and *M. purpureus* are mainly used for food in Japan, Taiwan, and China, respectively. It has been reported that the gene encoding citrinin, a mycotoxin causing renal toxicity, is functional in some strains of *M. purpureus* and *M. ruber* [81,82]. We recently conducted whole-genome analysis of these three strains of *Monascus* fungi using a next generation sequencer and demonstrated that *M. pilosus* was unable to generate citrinin [83]. This finding indicates that, of the three commercially used *Monascus* fungi, only *M. pilosus* does not generate the mycotoxin, citrinin. Consequently, based on its long history and the results of recent studies, red yeast rice is a safe material as long as its prepared and used properly.

## 5. Conclusions

In paragraph 1, we introduced that red yeast rice has been a prized medicinal food in East Asia for about 1500 years, and has been widely used as a food and pigment in recent years. In paragraph 2, we introduced the metabolites of red yeast rice, mainly polyketide compounds. These ingredients vary depending on the type of Monascus and the culture/manufacturing method, but even today, red yeast rice produced by tradi-tional solid fermentation is widely used as a health food material and we showed the changes in the amounts of the typical components during the solid-state fermentation process. In the future, it is expected to develop red yeast rice materials that has more beneficial balances of ingredients in the safe ancient manufacturing method.

In Section 3, we introduced mainly introduced the findings on the usefulness of red yeast rice on lipid metabolism and circulatory system. The data from our animal studies described in Section 3.1 and Section 3.2 suggest that in addition to monacolin K, other compo-nents of red yeast rice may improve lipid metabolism. However, it is a future issue to conduct experiments with more appropriate controls to clarify in more detail whether the effects other than LDL-cholesterol lowering effect are due to the red yeast rice component alone or the combination of monacolin K and red yeast rice components. In addition, the findings of the effects of statins, pigments, and GABA on the circulatory system as typical components were introduced in Section 3.3, but the involvement of various components reported to contain in red yeast rice, such as sterols, decalin compounds, the derivatives, flavonoids, lignans, coumarins, terpenoids, polysaccha-rides and phenolic acids is unknown [23]. Further elucidations of the relationship be-tween each of components of red yeast rice are expected.

Here, we introduced the typical lipid metabolism and effects of monacolin K (lovastatin) on the lipid metabolism and circulatory system, but recent reports have shown that monacolin K is also useful for the treatment of neurological disorders, cancer, etc. and these studies are also being highlighted [84]. Furthermore, various functionalities such as anti-cancer effect, neuroprotective effect, liver protective effect, osteoporosis improving effect, anti-diabetic effect, anti-obesity effect, anti-fatigue ef-fect, and anti-inflammatory effect have been reported for red yeast rice [23]. It has been suggested that various components other than monacolin K are involved in these actions in a complex manner. In the future, it is expected that evidence will be accumulated and will help improve the quality of life in a wide range beyond the prevention of lifestyle-related diseases.

In conclusion, red yeast rice is promising as a functional food material that can maintain and improve health in addition to preventing lifestyle related diseases. As a food material, it has high medicinal properties, various functions and safety. It is expected that new values will be elucidated in the future.

## Figures and Tables

**Table 1:** Typical *Monascus* fungi and their isolation source.

Strain	Source
*M. purpureus*	Red yeast rice, malted rice (*miquzi in Chinese)* (China, Korea, Taiwan)
*M. anka*	Red yeast rice (Taiwan), malted rice of red *nyufu*
*M. anka var. rubellus*	Lees from the making process of red *Laojiu* (Chinese alcoholic beverage)
*M. barkeri*	Malted rice for *samutu-syu* (Chinese red alcoholic beverage)
*M. albidus*	*Chantofu* (Shanghai)
*M. araneosus*	Malted rice for *gaoliangilu* (Chinese distilled spirit, Northeastern China)
*M. furiginosus*	Malted rice (Guizhou Province, China)
*M. major*	Malted rice (Fuzhou, China)
*M. albidus var. glaber*	Malted rice (Fuzhou, China)
*M. pilosus*	Malted rice for *gaoliangilu* (Fengtian, China)
*M. rubropanctatus*	Powdered malted rice for medicinal wine (Incheon, Korea)
*M. pubigerus*	Malted rice for *gaoliangilu* (Liaoyang, China)
*M. rubinosus*	Malted rice (Guangdong Province)
*M. serorubescens*	Red *funyu* (Hong Kong)
*M. vitreus*	Red *funyu* (Hong Kong)
*M. kaoliang*	Malted rice for *gaoliangilu* (Taiwan)
*M. ruber*	Silage, soil, rotten fruit etc.
*M. paxi*	Dead branch and leaves of plants

**Table 2:** Representative *Monascus* fungi preserved at the Biological Resource Center (NBRC), NITE in Japan.

Strain
*M. anka* Nakazawa et Sato NBRC 4478
*M. purpreus* Went NBRC 4513
*M. anka* Nakazawa et Sato (T. Hasegawa) NBRC 6540
*M. major* Sato NBRC 4485
*M. ruber* van Tieghem NBRC 4492
*M. pubigerus* Sato NBRC 4521
*M. araneosus* Sato NBRC 4482
*M. rubiginosus* Sato NBRC 4484
*M. anka* var. *rubellus* Sato NBRC 5965
*M. ruber* van Tieghem (S. Udagawa) NBRC 9203
*M. pilosus* Sato (FAT) NBRC 4520
*M. fuliginosus* Sato NBRC 4483
*M. pilosus* Sato NBRC 4480
*M. paxii* Lingelsheim NBRC 8201
*M. vitreus* Sato NBRC 4532
*M. vitreus* Sato (J. Nicot) NBRC 7537
*M. albidus* Sato NBRC 4489
*M. anka* var. *rubellus* Sato (H. Iizuka) NBRC 6085
*M. serorubescens* Sato NBRC 4487
*M. albidus* var. *glaber* Sato NBRC 4486
*M. serorubescens* Sato (FAT) NBRC 4525

**Table 3:** Pharmacokinetic parameters of the lactone form and acid form of monacolin K in plasma after single administration of purified monacolin K or red yeast rice (40 mg/kg monacolin K or dose of red yeast rice equivalent to 40 mg/kg monacolin K). Peak analysis was performed according to the reference method [44].

PK Parameter of Monacolin K Lactone						PK Parameter Of Monacolin K Hydroxy Acid					
Monacolin K Administration Group						Monacolin K Administration Group					
PK Parameter	101	102	103	Mean	S.D.	PK Parameter	101	102	103	Mean	S.D.
t_1/2_ (h)	N.C.	N.C.	N.C.	-	-	t_1/2_ (h)	2.7	1.7	N.C.	2.2	-
T_max_ (h)	1.0	1.0	N.C.	1.0	-	T_max_ (h)	1.0	1.0	2.0	1.3	0.6
C_max_ (ng/mL)	3.11	1.85	N.C.	2.48	-	C_max_ (ng/mL)	72.6	113	55.5	80.4	29.5
AUC_0–4h_ (ng·h/mL)	3.11	1.85	N.C.	2.48	-	AUC_0–4h_ (ng·h/mL)	143	274	153	190	73
Red yeast rice administration group						Red yeast rice administration group					
PK parameter	201	202	203	Mean	S.D.	PK parameter	201	202	203	Mean	S.D.
t_1/2_ (h)	N.C.	N.C.	N.C.	-	-	t_1/2_ (h)	1.4	1.1	0.98	1.1	0.2
T_max_ (h)	1.0	1.0	1.0	1.0	0.0	T_max_ (h)	1.0	1.0	1.0	1.0	0.0
C_max_ (ng/mL)	4.32	4.03	3.54	3.96	0.39	C_max_ (ng/mL)	254	349	406	336	77
AUC_0–4h_ (ng·h/mL)	7.20	5.22	6.46	6.29	1.00	AUC_0–4h_ (ng·h/mL)	457	470	632	520	98
						S.D.: Standard deviation					

**Table 4:** Actions of monacolin K (lovastatin) on circulatory system and related fields.

Evaluation Target	Study Subjects or Study Materials	Effect of Monacolin K	Ref.
Atheroma formation	Rabbits fed on a high-lipid diet	Decrease in number of plaques in the aorta and lung artery	[52]
	Patients with hypercholesterolemia	Inhibition of platelet aggregation, macrophage foam cell formation and LDL oxidation	[53]
	Human umbilical vein endothelial cells (HUVECs)	Inhibition of NF-KB activation by CRP	[54]
Blood circulation	Hypertensive model mice	Increase in renal blood flow	[55]
	Several studies using experimental animals	Increase in cerebral blood flow and cerebral vasomotor response	[56]
NOS signaling pathway	Cardiac myocytes	Increase of IL-1 induced production of nitrite (Increase in NOS expression and NO production mediated by Rho inhibition)	[61]
	Squamous epithelial cancer cells	Induction of expression of Rho family proteins	[62]
	Mesenchymal stem cells derived from rat bone marrow	Activation (phosphorylation) of AI3K/Akt pathway and MEK 1/2 pathway	[63]

## Data Availability

Not applicable.

## Sample Availability

Samples of the compounds are not available from the authors.
